# Lipid moieties on lipoproteins of commensal and non-commensal staphylococci induce differential immune responses

**DOI:** 10.1038/s41467-017-02234-4

**Published:** 2017-12-21

**Authors:** Minh-Thu Nguyen, Julia Uebele, Nimerta Kumari, Hiroshi Nakayama, Lena Peter, Olga Ticha, Anne-Kathrin Woischnig, Mathias Schmaler, Nina Khanna, Naoshi Dohmae, Bok Luel Lee, Isabelle Bekeredjian-Ding, Friedrich Götz

**Affiliations:** 1 Microbial Genetics, Interfaculty Institute of Microbiology and Infection Medicine Tübingen (IMIT), University of Tübingen, Tübingen, 72076 Germany; 2 Paul-Ehrlich-Institute, Federal Regulatory Agency for Vaccines and Biomedicines, Langen, 63225 Germany; 3 Biomolecular Characterization Unit, RIKEN Center for Sustainable Resource Science, Saitama, 351-0198 Japan; 4 Laboratory of Infection Biology, Department of Biomedicine, University Hospital Basel, Basel, CH-4031 Switzerland; 5 National Research Laboratory of Defense Proteins, College of Pharmacy, Pusan National University, Pusan, 609-735 South Korea; 6 School of Biological and Food Technology, Hanoi University of Science and Technology, Hanoi, 1000 Vietnam

## Abstract

Lipoproteins (Lpp) of Gram-positive bacteria are major players in alerting our immune system. Here, we show that the TLR2 response induced by commensal species *Staphylococcus aureus* and *Staphylococcus epidermidis* is almost ten times lower than that induced by noncommensal *Staphylococcus carnosus*, and this is at least partially due to their different modifications of the Lpp lipid moieties. The N terminus of the lipid moiety is acylated with a long-chain fatty acid (C17) in *S. aureus* and *S. epidermidis*, while it is acylated with a short-chain fatty acid (C2) in *S. carnosus*. The long-chain *N*-acylated Lpp, recognized by TLR2–TLR1 receptors, silences innate and adaptive immune responses, while the short-chain *N*-acetylated Lpp, recognized by TLR2–TLR6 receptors, boosts it.

## Abstract (web summary)

The Lpp lipoproteins of staphylococci trigger a TLR2-dependent immune response. Here, the authors show that commensal species (*S. aureus, S. epidermidis*) induce a less-intense TLR2 response than non-commensal species (*S. carnosus*) due to differential modification of the Lpp lipid moieties.

## Introduction

Bacterial lipoproteins (Lpp) possess a lipid moiety at their N terminus that enables their anchorage to the bacterial membranes^1, 2^. The maturation and processing of Lpp involve three enzymes: diacylglyceryl transferase (Lgt), lipoprotein signal peptidase (Lsp), and an apolipoprotein *N*-acyltransferase (Lnt). In *Escherichia coli*, all three enzymes are essential and localized in the cytoplasmic membrane^3^. In Gram-positive bacteria that lack the outer membrane, Lpp are anchored in the outer leaflet of the cytoplasmic membrane where they play a supportive role in uptake of nutrients, acquisition of essential ions, and maintaining metabolic activity and bacterial survival in infection^4^. In Gram-positive bacteria, Lpp control pathogenicity and immunity^5–7^.

Lpp can be separated into two functional units: the protein part is responsible for the metabolic function, while the lipid moiety anchors the protein in the membrane. However, this lipid structure, unique to bacteria, also acts as “danger signal” that alerts the innate immune system via activation of pattern recognition receptors (PRR). Thus, Lpp and lipopeptides represent important microbe-associated molecular patterns (MAMPs) that are recognized by the innate immune system, which represents the first line of defense against invasive infectious pathogens^8, 9^. The essential receptor for recognition of Lpp or synthetic lipopeptides is the Toll-like receptor (TLR)-2^10^. The lipid structure, namely the degree of acylation at the lipid moiety, can be discriminated by additional receptors that form heterodimers with TLR2: triacylated Lpp are recognized by the TLR2/TLR1^11^ and diacylated Lpp by the TLR2/TLR6 heterodimer^12–14^.

The signal transduction from Lpp-bound TLR2 (TLR1 or TLR6) heterodimer to the activation of the nuclear factor NF-kappaB involves a cascade of phosphorylation events^15^. The TIR domain-containing adapters MyD88 and TRIF are the first interaction partners and central adapter molecules in TLR2–TLR1/6-triggered signaling which is mediated by downstream activation of IRAK4^16^ and subsequent translocation of NF-kappaB into the nucleus. This enables transcription and synthesis of proinflammatory cytokines and chemokines^16–18^.

Since the number of acyl residues at the lipid moiety was shown to be crucial for their recognition via TLR2/1 or TLR2/6, it is important to know which bacteria produce di- or triacylated Lpp. To date, it is known that Gram-negative bacteria and also some high-GC Gram-positive bacteria such as mycobacteria and streptomycetes produce triacylated Lpp^19^. These bacteria encode an apolipoprotein *N*-acyltransferase (Lnt) homologous to that in *E. coli*. The membrane-bound Lnt acylates the free N terminus of the cysteine residues, thus forming the triacylated lipid moiety^20^. However, in low GC Gram-positive bacteria (*Firmicutes* phylum), the situation is much less clear because up to now, an enzyme with apolipoprotein *N*-acyltransferase function (e.g., Lnt) has not been identified.

Indeed, in certain bacterial species such as *Mycoplasma*, only diacylated Lpp are produced, such as the widely used macrophage-activating lipopeptide-2 (MALP-2)^14, 21^. As in *Staphylococcus aureus,* there was also no Lnt homolog identified, and it was assumed that in this bacterial species, Lpp are only diacylated like in *Mycoplasma* ^22^. However, Kurokawa and colleagues show that in *S. aureus,* the 33 -kDa Lpp SitC, which is considered a model Lpp because of its abundance in staphylococci, is triacylated and acts as a TLR2 ligand^23, 24^.

In principle, it should not matter whether the Lpp are di- or triacylated, because TLR2/1 or TLR2/6 heterodimers enable recognition of both Lpp types and, in both cases, this triggers NF-kappaB activation. However, recent reports have provided evidence that there are profound differences in the local immune response depending on whether the skin is exposed to di- or triacylated Lpp^25^. Exclusively diacylated Lpp were capable of suppressing immune responses through interleukin-6 (IL-6)-dependent induction of granulocytic and monocytic myeloid-derived suppressor cells. However, since these in vivo observations were mainly based on experimental treatments using synthetic Lpp, we asked whether such effects could also be mediated by commensal or noncommensal bacteria.

Here, we demonstrate that Lpp of the two commensal staphylococcal species, namely *S. aureus* and *Staphylococcus epidermidis*, tether the N terminus of *S*-(diacyl-glyceryl) cysteine residue of the lipid moiety with a long-chain acyl group (heptadecanoyl fatty acid). This lipid structure dampens the immune response. In the noncommensal species,* Staphylococcus carnosus*, the N terminus of the lipid moiety carries only a short-chain (acetyl) fatty acid, which induces a stronger innate and adaptive immune response.

## Results

### *S. carnosus* and *S. aureus* differ in immune stimulatory activity

**Fig. 1:**
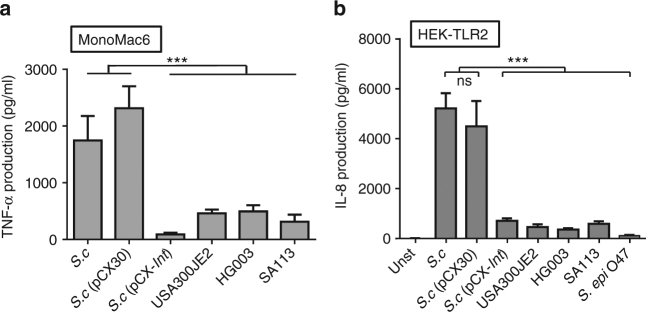
Induction of cytokines by MonoMac6 and HEK-TLR2 cells infected by different staphylococci strains. (**a**) TNF-α production (pg/ml) after 4-h stimulation of MonoMac6 cells with different staphylococcal strains (MOI 30). (**b**) IL-8 production (pg/ml) after 18-h stimulation of permanent TLR2-transfected HEK cells with different strains (MOI 10). For the infection, the staphylococcal strains were cultured in TSB plus 0.25% xylose for 15 h and then washed two times with DMEM/F. The experiments in triplicate were conducted at least two times. Error bars indicate standard error (SD). Statistical significances were calculated by using Student’s *t* tests or analysis of variance (ANOVA): not significant *P* > 0.05, *P < 0.05, **P < 0.01, ***P < 0.001. *S.c*, *S. carnosus*; *S.c* (pCX30) plasmid without insert; *S.c* (pCX-*lnt*) plasmid encoding *E. coli*-specific *lnt* gene; USA300JE2, HG003, and SA113 represent different *S. aureus* strains, and *S. epidermidis* O47

When MM6 cells were stimulated with different staphylococcal strains and species, we observed important differences in their immune stimulatory potential. Notably, the *S. carnosus* parent strain TM300 induced a >4× higher TNF-α production than the three *S. aureus* strains USA300JE2, HG003, and SA113 (Fig. 1a). Since, on the one hand, MM6 cells are responsive to a broad panel of agonists for PRR (e.g., TLR2, TLR4, TLR5, or Nod) and to adjuvants derived thereof^26, 27^, the high stimulatory activity of *S. carnosus* could result from recognition of a multitude of MAMPs. On the other hand, MM6 is unresponsive to TLR3, TLR7, TLR8, or TLR9 ligands and it is, therefore, unlikely that bacterial nucleic acids are responsible for the observed differences in immune stimulation. We, therefore, hypothesized that chemical differences in Lpp or peptidoglycan, which are recognized by TLR2 and Nod2, respectively, lead to differential immune recognition. Considering that TLR2 is a surface receptor and, thus, easily accessible and that peptidoglycan needs to gain access to its cytoplasmic receptors, Lpp were deemed the more likely candidates. To identify the MAMP responsible for the difference in immune stimulation, we repeated the experiment in HEK293 and TLR2-transfected HEK293 cells with the same stimulatory conditions. Untransfected HEK293 cells were not responsive to stimulation. However, in HEK-TLR2, we obtained the same pattern as that observed in MM6 cells: *S. carnosus* induced >10× higher IL-8 production than the three *S. aureus* strains and *S. epidermidis* O47 (Fig. 1b). These results indicate that TLR2-active Lpp are responsible for the differences in immune stimulatory activity. To verify this assumption, SitC, an abundant Lpp in staphylococci was expressed with a His tag and purified from both species and used for immune stimulation.

### SitC from *S. carnosus* shows a higher TLR2 response than that of *S. aureus*

**Fig. 2:**
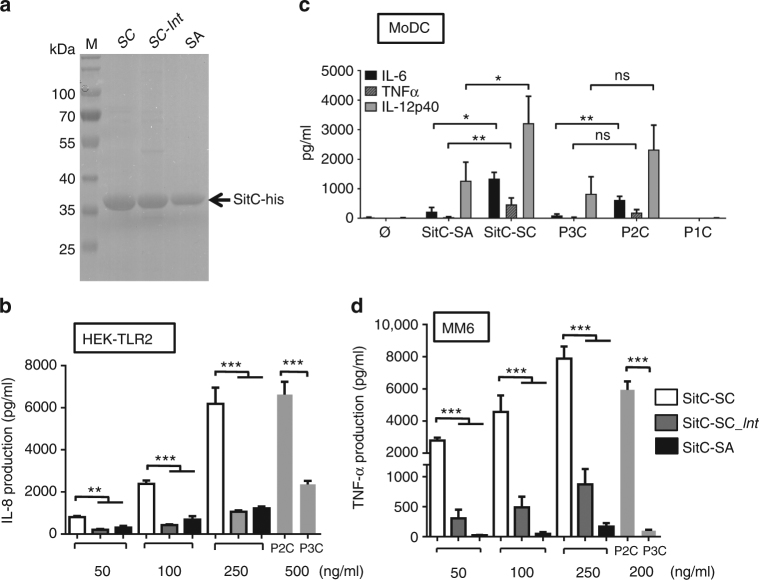
Purified SitC-SC induced ten times higher TLR2 signaling than SitC-SA. (**a**) The Coomassie blue-stained SDS page of purified SitC from *S. carnosus* (SitC-SC)*, S. carnosus* pCX*-lnt* (SitC-SC*lnt*), and *S. aureus* SA113 (SitC-SA); M, size standard. (**b**) IL-8 production (pg/ml) after 18-h stimulation of permanent TLR2-transfected HEK cells with purified SitC from *S. carnosus* (SitC-SC), *S. carnosus* pCX*-lnt* (SitC-SC*lnt*), and *S. aureus* SA113 (SitC-SA) with different concentrations (50, 100, and 250 ng/ml). For positive controls, synthetic Lpp Pam_2_CSK_4_ (P2C) and Pam_3_CSK_4_ (P3C) were applied in the same concentration (500 ng/ml). The experiments in triplicate were conducted at least three times. Error bars indicate standard error. Statistical significances were calculated by using Student’s *t* tests or analysis of variance (ANOVA): not significant *P* > 0.05, *P  < 0.05, **P < 0.01, ***P < 0.001. (**c**) Monocyte-derived dendritic cells (MoDC) were left unstimulated (Ø) or stimulated with 0.1 µg/ml SitC-SC or SitC-SA and 0.1 μg/ml P2C, P3C, or nonstimulatory P1C, respectively; cytokines in the cell-free supernatants were measured after 16 h by ELISA. Levels of IL-6, TNF, and IL-12p40 are displayed as the mean of *n* = 8 independent donors ± SEM. Statistical significance was calculated using the Wilcoxon matched-pairs signed rank test. *P < 0.05, **P < 0.01. (**d**) TNF production (pg/ml) after 4-h stimulation of MonoMac6 (MM6) with purified SitC from *S. carnosus* (SitC-SC), *S.carnosus* pCX*-lnt* (SitC-SC*lnt*), and *S. aureus* SA113 (SitC-SA) with different concentrations (50, 100, and 250 ng/ml). For positive controls, P2C and P3C were applied in the same concentration (200 ng/ml). The experiments in triplicate were conducted at least three times. Error bars indicate standard error. Statistical significances were calculated by using Student’s *t* tests or analysis of variance (ANOVA): not significant *P* > 0.05, *P < 0.05, **P < 0.01, ***P < 0.001

Previously, SitC has been described as one of the most prominent Lpp in *S. aureus* ^22^. It was originally referred to SitC because its protein sequence shares 77% identity to SitC of *S. epidermidis* where it was originally described as an iron transporter^28^. Later, it turned out that in *S. aureus*, SitC is involved in manganese (Mn) transport and it was also referred to as MntC^29^. The *sitC* gene was fused to a 3′ His tag, cloned into a xylose-inducible vector pTX-*sitC*-his, and expressed in *S. carnosus* TM300 and *S. aureus* SA113. From these two strains, SitC was extracted from the membrane fraction and purified via Ni-NTA chromatography. The two SitC lipoprotein preparations display a single band at 36 kDa in SDS-PAGE (Fig. 2a). As we express the same Lpp in various strains/species, we expect that folding and stability are the same. Equal amounts of purified SitC-his from both strains were used to stimulate HEK-TLR2 cells at varying concentrations (50, 100, and 250 ng/ml). Both SitC-his Lpp induced TLR2-mediated IL-8 secretion in a dose-dependent manner (Fig. 2b). However, SitC-SC (isolated from *S. carnosus*) induced almost tenfold higher IL-8 production than SitC-SA (isolated from *S. aureus*). Importantly, the synthetic Lpp, P2C Pam_2_CSK_4_, a synthetic dipalmitoylated lipopeptide that mimics the acylated amino terminus of bacterial lipoproteins, and P3C (Pam_3_CSK_4_, synthetic tripalmitoylated lipopeptide) (0.5 μg) also displayed an almost tenfold difference in IL-8 induction. In these synthetic Lpp, the diacyl-glyceryl residue is palmitoylated. The only difference lies in the modification of the N terminus of the *S*-(diacyl-glyceryl) cysteine residue: P3C is *N*-palmitoylated, while P2C is unmodified. These results are a first hint that the marked difference in TLR2 activation between *S. aureus* and *S. carnosus* could originate from species-specific differences in the lipidation structures of the Lpp.

To confirm the results obtained in HEK-TLR2 to more relevant cell systems, we investigated cytokine profiles of human cells. Using monocyte-derived dendritic cells (MoDC), we observed similar differences as in HEK-TLR2. Induction of the proinflammatory cytokines IL-6 and TNF and the Th1 polarizing cytokine IL-12p40 was higher when MoDC were stimulated with SitC-SC than with SitC-SA (IL-12p40: threefold, IL-6: fivefold, and TNF: 10-fold increase); similarly, the synthetic lipopeptide P2C was more potent than P3C (Fig. 2c). The results in MM6 cells show the same tendency, e.g., higher secretion of TNF with SitC-SC stimulation than with SitC-SA (Fig. 2d). The results confirm that Lpp of *S. carnosus* and *S. aureus* differ in their immune stimulatory potential. Differences in the lipid structure could account for the differences in cytokine secretion levels. Most likely, Lpp in *S. carnosus* are diacylated, while those of *S. aureus* are triacylated. Notably, stimulation with the synthetic Lpp resulted in analogous differences in immune stimulation. We concluded that *S. carnosus* lacks the amide-bound fatty acid at the N terminus of cysteine as in P2C. If this was the case expression of the *E. coli*-specific apolipoprotein *N*-acyltransferase gene, *lnt* in *S. carnosus* should decrease immune stimulation to the levels seen with *S. aureus*.

### Cloning of the *E. coli*-specific *lnt* in *S. carnosus* decreased TLR2-dependent signaling

**Fig. 3:**
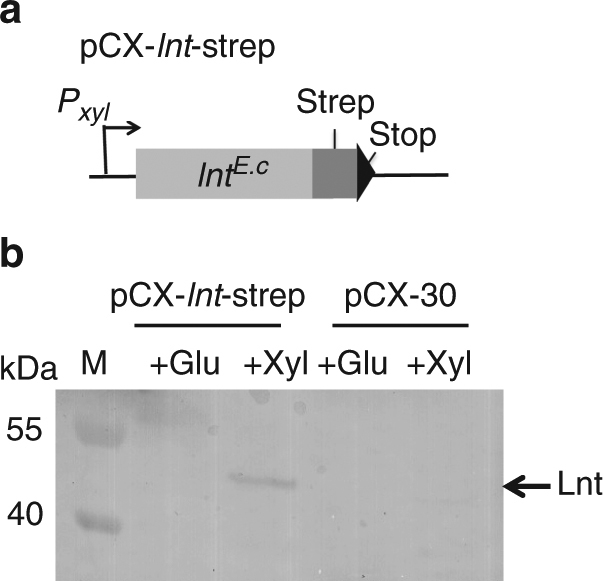
Expression of *E. coli*-specific *lnt* gene in *S. carnosus*. (**a**) Plasmid construct: in pCX-*lnt*-strep, the *E. coli*-derived *lnt* carries a strep-tag sequence and two 3′ stop codons; its expression is xylose inducible and glucose repressible. (**b**) Western blot with membrane fractions generated from *S. carnosus* (pCX-*lnt*-strep) and *S. carnosus* (pCX30) cultivated in B medium supplemented with either 0.25% glucose (causes *lnt* repression) or 0.25% xylose (causes *lnt* induction); Lnt was targeted with α-strep-tag antibody; M, size standard

The *E. coli*-specific *lnt* gene supplied with a 3′ strep tag was cloned into *S. carnosus* using the pCX-*lnt*-strep vector in which *lnt* expression is repressed by glucose but induced by xylose^30^ (Fig. 3a). In *E. coli*, Lnt is an integral membrane protein with a periplasmic loop containing the active site^31^. Western blot (alpha-strepAB) results confirmed that in *S. carnosus*, Lnt (mass 57 kDa) was localized in the membrane fraction and could only be detected when the transformed cells were grown in the presence of xylose (0.25%) but not glucose or in both cases in the vector control without *lnt* insert *S. carnosus* (pCX30) (Fig. 3b). Notably, in the *S. carnosus,* mutant expression of Lnt was low but this is also the case in *E. coli* where its abundance was calculated with ~100–200 molecules/cell^32, 33^. The most important result was that *S. carnosus* (pCX-*lnt*) induced ten times less TNF-α and IL-8 production than *S. carnosus* wild type and *S. carnosus* (pCX30) (Fig. 1a, b). Thus, expression of Lnt reduced the immune stimulatory effect to the level seen with the *S. aureus* strains. Moreover, SitC purified from *S. carnosus* (pCX-*lnt*), termed as SitC-SC*lnt*, also triggered tenfold less IL-8 and TNF-α production compared to SitC-SC upon stimulation of MM6 and HEK-TLR2 cells (Fig. 2b, d). This result supported the hypothesis that the *N*-acylation of Lpp in *S. aureus* strains prevents TLR2-dependent immune stimulation when compared to Lpp of *S. carnosus*, in which we assumed the N terminus to be unmodified. To prove this hypothesis, the lipid structure of purified SitC extracted from *S. carnosus* and *S. carnosus* (pCX-*lnt*) was analyzed.

### Lpps from *S. carnosus* are aminoacylated with a short-chain fatty acid (C2) while those from *S. carnosus* (pCX-*lnt*), *S. aureus,* and *S. epidermidis* with a long-chain fatty acid (C17)

**Fig. 4:**
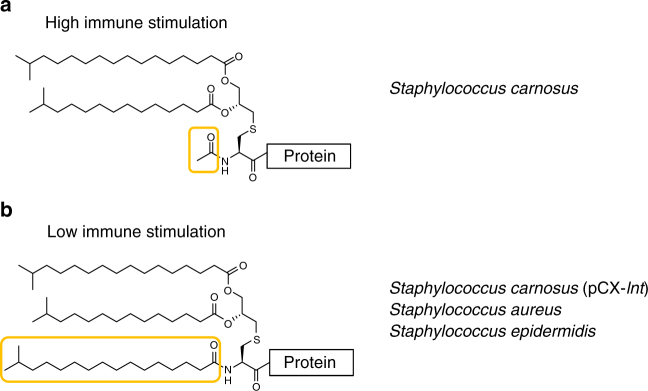
The *N*-acetylated and *N*-long-chain-acylated triacyl structures of staphylococcal Lpp. The *N*-acetylation (**a**) is detected in *S. carnosus*, while the *N*-long-chain acylation (**b**) is found in *S. aureus*, *S. epidermidis*, and *S. carnosus* (pCX-*lnt*). Representative structures of each type are shown. The long-chain acylation at the α-amino group of Lpp varies in length in *S. aureus* and *S. epidermidis*. In contrast, only heptadecanoyl group is detected in *S. carnosus* (pCX-*lnt*). The sn-1 and sn-2 positions are O-acylated with various lengths of fatty acids (15:0–21:0) and exclusively *O*-pentadecanoic acid (15:0) in all the staphylococcal Lpps examined in this study

In order to demonstrate that the immune stimulatory activity is directly related to *N*-acylation of Lpp, we analyzed their lipidation by mass spectrometry (MS) and tandem mass spectrometry (MS/MS). The representative structures of the staphylococcal Lpp determined in this study are summarized in Fig. 4. First, the His-tagged SitC protein isolated from *S. carnosus* (SitC-SC) was subjected to SDS-PAGE and in-gel trypsin digestion. The resulting digest was analyzed by matrix-assisted laser desorption/ionization time-of-flight (MALDI TOF) MS and nanoflow liquid chromatography (nLC)–MS. The MALDI mass spectrum of the digest of SitC-SC shows ions with 14-Da mass differences between *m/z* 1058.8 and 1184.8 (Supplementary Fig. 1a), which can correspond to either diacyl (between 32:0 and 39:0; total numbers of carbon atoms and double bonds in their fatty acyl groups) or triacyl (between 31:0 and 38:0) lipopeptides. The accurate mass values obtained by LC–MS unexpectedly determined that they were triacyl forms (Supplementary Fig. 2). To obtain direct evidence of *N*-acylation of the triacylated SitC-SC, we then performed MS/MS analyses. nLC–MS/MS spectrum of each major lipopeptide ion demonstrated that the amino group of SitC-SC was acetylated (2:0) (Supplementary Fig. 3). The MS/MS spectra also allowed us to determine that the *sn*-1 position was *O*-acylated with fatty acids of various lengths (between 15:0 and 20:0), while the *sn*-2 position in *S*-glyceryl moiety was exclusively *O*-pentadecanoylated (15:0), which is consistent with the composition of membrane lipids in *S. aureus* ^34, 35^. Next, we analyzed the SitC protein prepared from *S. carnosus* (pCX-*lnt*) (pTX30-*sitC*-his). The MALDI TOF mass spectrum of SitC-SC*lnt* shows ions between *m/z* 1297.0 and 1381.0 corresponding to triacyl (between 47:0 and 55:0) lipopeptides (Supplementary Fig. 1b). Further, MS/MS analyses demonstrated that the N terminus of SitC-SC*lnt* was heptadecanoylated at the amino group and *O*-acylated with similar fatty acids to those in SitC-SC, as shown in Supplementary Fig. 4. Note that no ion corresponding to *N-*acetyl forms could be detected by MALDI TOF MS although one would expect to detect a mixture of both the lipidations in the bacterium. Then, we confirmed our previous results on lipidation in *S. aureus* and *S. epidermidis* ^23, 36^; the tagged SitC protein expressed in both *S. aureus* (SitC-SA) and *S. epidermidis* (SitC-SE) were triacyl structures with varying lengths of acylation (Supplementary Fig. 1c, d). The most abundant lipidation forms for SitC-SC*lnt*, SitC-SA, and SitC-SE were 49, 51, and 53, respectively (Supplementary Fig. 1b–d), which probably reflects the difference in membrane lipid compositions of these three strains.

To further understand whether Lpp in *S. carnosus* are generally *N*-acetylated, we performed LC–MS/MS analysis of another Lpp, Lpl1, whose amino acid sequence is significantly different from that of SitC. Lpl1 is the first Lpp encoded by the tandem *lpl*-gene cluster in *S. aureus* USA300^37^. We analyzed the *S. aureus* Lpl1-his protein expressed by *S. carnosus* (Lpl1-SC) and that in *S. aureus* (Lpl1-SA) by LC–MS/MS. The accurate mass measurement and MS/MS analysis of Lpl1-SC provided unequivocal evidence that the N terminus of Lpl1-SC was also *N*-acetylated (2:0) and that the *S*-glyceryl moiety was *O*-acylated with fatty acids (one of 17:0–21:0 at *sn*-1 and 15:0 at *sn*-2), which closely resembles the lipidation of SitC-SC (Supplementary Fig. 5). For Lpl1-SA, MS, and MS/MS, results of the in-gel digests revealed that the *N*-terminal lipopeptide of Lpl1-SA was *N*-long-chain-acylated triacyl one with various lengths of fatty acids as reported previously^36^ (Supplementary Fig. 6). These results collectively indicated that altering the aminoacylation from *N*-acetylation to *N*-long-chain acylation could reduce the stimulatory activity of staphylococcal lipoproteins on the host immune response.

### Short-chain *N*-acetylated SitC-SC triggered a TLR2/6 response while long-chain *N*-acylated SitC-SA a TLR2/1 response

**Fig. 5:**
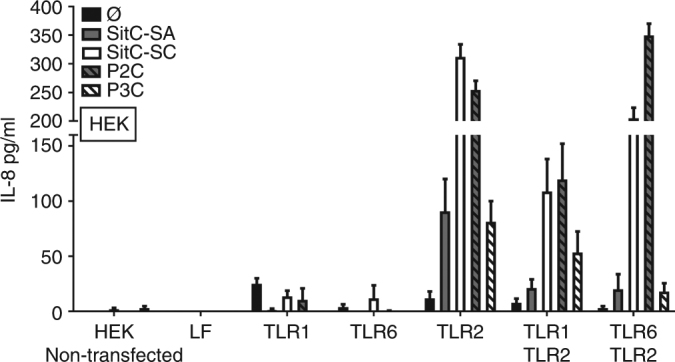
Cytokine response of HEK cells transfected with TLR1, 2, and 6 receptor cDNA to various TLR2 ligands. To assess differential recognition of di- and triacylated Lpp by TLR1/TLR2 and TLR6/TLR2 heterodimers in HEK293 cells, HEK293 cells were transfected with 90 ng of TLR1 or TLR6 or 100 ng of TLR2 cDNA alone or with low TLR2 cDNA (10 ng) together with 90 ng of TLR1 or TLR6, respectively. In all conditions, cells were either unstimulated (Ø), stimulated with 100 ng of SitC purified from *S. carnosus* (SitC-SC) or from *S. aureus* (SitC-SA), or with 100 ng of synthetic Lpp Pam_2_CSK_4_ (P2C) and Pam_3_CSK_4_ (P3C), respectively. IL-8 secretion was quantified by ELISA 18 h after stimulation and compared with untransfected HEK293 cells (left) or lipofectamine only (LF) as controls. The diagram shows the results obtained in three representative experiments performed in duplicates

HEK293 cells were transfected with pFLAG-CMV-1 expressing TLR1, TLR2, or TLR6 alone or in combination and were stimulated with SitC-SC, SitC-SA, P2C, and P3C, respectively. The chemokine IL-8 was used as a readout of the TLR-induced NF-κB activation (Fig. 5). HEK293 cells constitutively express low levels of endogenous TLRs including TLR1 and TLR6 but lack the expression of endogenous TLR2, TLR4, as well as other associated molecules including CD14 and MD2^38^. When we compared wild-type HEK293 cells and the TLR-transfected cells, the wild-type cells were unresponsive to any Lpp ligand, and stimulation of TLR1- and TLR6-transfected cells was negligible. By contrast, TLR2-transfected cells were highly responsive to P2C and SitC-SC and to a lesser extent to P3C and SitC-SA ligands. This finding indicates that there is sufficient endogenous expression of TLR1 and TLR6 in wild-type HEK293 cells to allow heterodimer formation with TLR2 (Fig. 5). Upon overexpression of TLR1 in TLR1 and TLR2 cotransfected cells, one would expect that recognition of P3C and SitC-SA would be strongly improved; however, P2C and SitC-SC remained superior to the triacylated ligands. We assume that the endogenous expression of TLR6 is sufficient to overrule TLR1 expression. Finally, TLR6 and TLR2 cotransfected cells were highly responsive to P2C and SitC-SC and displayed only very limited activity when exposed to P3C and SitC-SA. Again, this suggested that overexpression of TLR6 overrules TLR1/TLR2 responses. The results indicated that P2C and SitC-SC induce nearly comparable and high activation of TLR2/TLR6 heterodimers and that the short-chain *N*-acylated SitC-SC (the N terminus is only acetylated) behaves like the N-unmodified P2C (diacylated lipopeptide). Apparently, the short N-acetyl residue in SitC-SC is too small to function as a TLR2/1 agonist. The results further imply that TLR6/2 ligands dominate over TLR1/2 agonists with respect to induction of TLR2 signaling. The subsequent question was whether SitC-SC and SitC-SA also differ in their impact on the adaptive immune response.

### SitC-SC is a more potent inducer of Th1 responses than SitC-SA

**Fig. 6:**
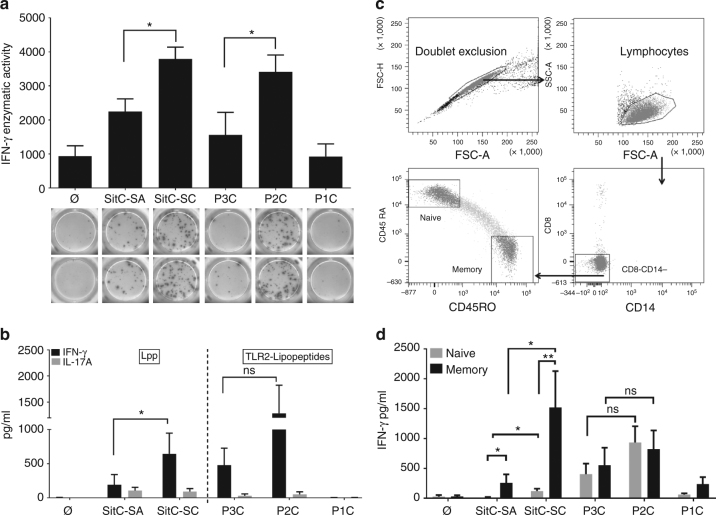
Purified SitC-SC induced a stronger Th1 cytokine response from MoDC/T cocultures than SitC-SA. (**a**) Activation of IFNγ-secreting T cells was measured in PBMC spiked with MoDC prestimulated with TLR2 ligands (0.1 μg/ml), i.e., SitC-SC, SitC-SA, P2C, P3C, or left unstimulated (Ø) or treated with P1C as controls. After 2.5-h stimulation, MoDC were added to freshly thawed autologous PBMC. IFNγ-secreting cells were quantified by ELISPOT after overnight incubation. The results are displayed as means of the enzymatic activity of *n* = 6 independent donors ±SEM (upper panel). The two-tailed Student’s *t* test was used to calculate significance (*P < 0.05). ELISPOT images from one representative experiment are shown in the lower panel. (**b**) MoDC were left unstimulated or stimulated with SitC derived from either *S. carnosus* (SitC-SC) or from *S. aureus* (SitC-SA) or with synthetic TLR2 ligands P3C, P2C, or P1C as control (1 μg/ml). Total CD4+ T cells were added to stimulated MoDC, MoDC/T cell cocultures incubated for 5 days before IFNγ and IL-17 secretion was quantified in the supernatants by ELISA. Donors with strong reactivity in the unstimulated and P1C conditions were excluded from the analysis. The graph depicts the results obtained from *n* = 6 independent donors. The Wilcoxon matched-pairs signed rank test was used for statistical analysis (*P < 0.05). (**c**) Sorting scheme for obtaining CD4+ naive (CD45RA+CD45RO−) and memory (CD45RA− CD45RO+) T cell fractions. (**d**) Sorted naive and memory T cell fractions were cocultured with MoDC in the presence and absence of SitC-SA, SitC-SC, or P3C, P2C, and P1C (0.1 μg/ml). After 5 days, T cell-derived IFNγ secretion was analyzed in the cellular supernatants. The graphs depict the results obtained from 12 independent donors as mean values ± SEM. Statistical analysis was performed using the Wilcoxon test (*P < 0.05; **P < 0.01)

To assess the effect of the TLR2-active component in SitC on T cell responses, we analyzed secretion of the T cell-derived cytokine IFNγ in PBMC stimulated with MoDC pretreated with the TLR2 ligands, e.g., SitC derived from *S. carnosus* (SitC-SC) or *S. aureus* (SitC-SA) or the synthetic TLR2 agonists P3C, P2C, or nonstimulatory, monoacylated P1C (Pam-Dhc-CSK4, synthetic monopalmitoylated lipopeptide) as control. Figure 6a shows the results obtained by ELISPOT analysis. Notably, in the presence of triacylated Lpp (SitC-SA and P3C), the IFNγ response was lower than with diacylated P2C and the N-acetylated SitC-SC.

To confirm that IFNγ secretion originates from Th1 cells, we next retrieved to MoDC cocultures with purified total CD4+ T cells. MoDC were stimulated with SitC protein, TLR2-active lipopeptides (P2C, P3C), or P1C and cocultured with autologous CD4+ T cells for 5 days. Approximately fivefold higher secretion levels of the Th1 lead cytokine IFNγ were detected in the supernatants of CD4+ T cells stimulated with MoDC challenged with SitC-SC when compared to SitC-SA (Fig. 6b). Similarly, P3C elicited higher levels of IFNγ secretion when compared to P2C. By contrast, secretion of IL-17A was comparable in SitC-SC and SitC-SA-stimulated cultures but secretion levels remained low (≤105 pg/ml) and IL-17 was nearly absent in conditions with P3C and P2C.

Finally, we separated naive from memory CD4+ T cell fractions to investigate whether one of these subpopulations is more prone to respond to SitC or TLR2 ligands (Fig. 6c). IFNγ responses were measured in supernatants from cocultures with MoDC stimulated with SitC-SC, SitC-SA, or P3C and P2C or unstimulated with P1C controls. CD4+ memory T cells were highly responsive to SitC-SC but SitC-SC only elicited very low levels of IFNγ in naive T cells (12-fold difference) (Fig. 6d). The response to SitC-SA was significantly lower in both memory and naive cell fractions (6- and 10-fold difference to SitC-SC, respectively). Of note, in contrast to SitC, naïve and memory T cell fractions were equally responsive to P3C and P2C (Fig. 6d).

Altogether, SitC-SC is superior to SitC-SA in regard to promoting a Th1 response; this is particularly reflected by its ability to trigger an IFNγ response in CD4+ memory T cells. Lipid modifications in SA might, thus, suppress this type of T cell memory, which is generally regarded as protective.

### *S. carnosus* (pCX-*lnt*) induced lower TNF-α cytokine in vivo than parent *S. carnosus*

**Fig. 7:**
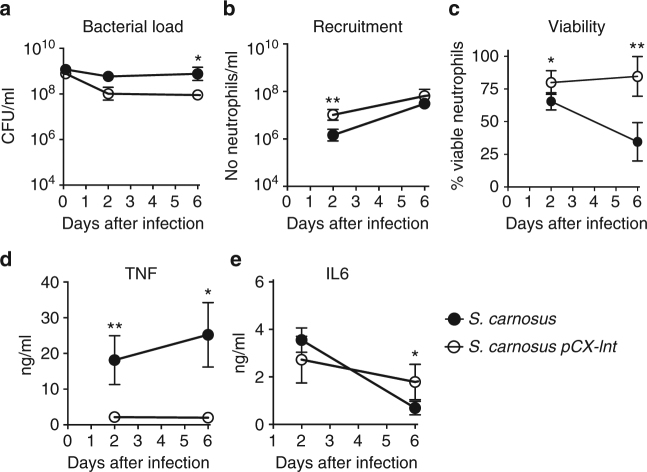
Mice and tissue cage infection model. Tissue cages were implanted in C57BL/6 mice and infected percutanously with around 10^9^ *S. carnosus* (filled) and *S. carnosus lnt* (open). (**a**) Bacteria and (**b**) neutrophils were counted, (**c**) neutrophil viability was determined, and (**d**) TNF and (**e**) IL-6 concentrations were measured in tissue cage fluid (TCF) during 6 days. *P ≤ 0.05 and **P ≤ 0.01, analyzed using Mann–Whitney test. Data show mean ± s.d. of 5–6 mice in each group from two independent experiments

To test whether differences in the acylation of lipoproteins may change the immune responses of leukocytes including neutrophils and monocytes, we made use of the murine tissue cage infection model^39^. Since a homolog of *lnt* was not found in *S. aureus*, we used the *S. carnosus* and the *S. carnosus* strain expressing the *lnt* gene to compare their capacity to establish a perioperative infection of C57BL/6 mice. In the perioperative infection of C57BL/6 mice, around 10^9^ CFU of *S. carnosus* parent strain and *S. carnosus* (pCX-*lnt*) were injected directly into the tissue cages. CFU numbers of *S. carnosus* were stable until day 6 after infection (Fig. 7a). In contrast, CFU numbers of *S. carnosus* (pCX-*lnt*) decreased until day 6 and were significantly lower on day 6 compared with *S. carnosus* (Fig. 7a). The total number of neutrophils in tissue cage fluid (TCF) of mice infected with *S. carnosus* was lower than that in mice infected with *S. carnosus* (pCX-*lnt*) on day 2 (Fig. 7b), but neutrophil numbers on day 6 were comparable, suggesting that neutrophil numbers were not related to CFU numbers. Therefore, neutrophil viability and cytokine release were determined in TCF from mice infected with both strains. On day 2, neutrophil viability was reduced in TCF from mice infected with *S. carnosus* compared to *S. carnosus* (pCX-*lnt*) (Fig. 7c); an effect that was even more pronounced on day 6 after infection (Fig. 7c). In line with this observation, TNF-α concentrations were increased in TCF of mice infected with *S. carnosus* compared with *S. carnosus* (pCX-*lnt*) (Fig. 7d). IL-6 release was similar on day 2, but higher in TCF from mice infected with *S. carnosus* compared with mice infected with *S. carnosus* pCX-*lnt* on day 6 (Fig. 7e). Thus, the unmodified or short-chain (acetyl) modified N terminus of *S. carnosus* Lpp induced tremendous TNF release in recruited neutrophils, increased cell death, and impaired bacterial killing in implant-associated infections.

## Discussion

The skin is the organ most exposed to the environment; at the same time, it harbors highly diverse bacterial communities belonging to four major phyla: *Actinobacteria*, *Bacteroidetes*, *Firmicutes*, or *Proteobacteria* ^40^. Among the *Firmicutes*, staphylococci occupy a special position as many species are commensals of the skin of humans and animals. Each of the various areas within our skin, e.g., the moist, sebaceous, or dry sites represent niches for different staphylococcal species^41, 42^. It is apparent that host factors, immunological imprintment, and habits shape the composition of these microbial communities; vice versa, microbes present on the skin strongly impact the functions of human immunity^43^. It was the aim of this study to investigate the reciprocal influence between the microbiota and the host immune response.

Here, we investigated the immune response of representatives of three different staphylococcal species: *S. aureus*, *S. epidermidis*, and *S. carnosus*. *S. epidermidis* is the most common clinical isolate derived from the cutaneous microbiota. Formerly, it was regarded as a harmless commensal but due to its biofilm-forming ability, it emerged as a frequent cause of foreign-body-associated chronic infections^44–48^. *S. aureus* is also a skin commensal and its important niche is the anterior nares from where it can be isolated in more than one-third of the population^49^. However, in contrast to *S. epidermidis*, *S. aureus* is equipped to produce an arsenal of toxins that promote acute and sometimes fulminant infections^50–53^. Despite this battery of toxins, *S. aureus* is found on healthy human skin, acting as a commensal rather than a pathogen^54, 55^. In contrast to *S. aureus* and *S. epidermidis*, *S. carnosus* has the least pathogenic potential and it is not a commensal of human skin^56^; its original habitat remains undefined although it was speculated that it might originate from marine fish^57^.

Comparing the immune response of the two human skin commensals (*S. aureus* and *S. epidermidis*) with the noncommensal species *S. carnosus,* we were stricken by the fulminant immune response against *S. carnosus*, which induced almost 10-times more IL-6 with MM6 than *S. aureus* ^37^. At that time, it was not clear which of the MAMPs was responsible for this effect: RNA, DNA, peptidoglycan, Lpp, and all possible combinations were in question. Since in *S. aureus* lipidation of Lpp by the phosphatidylglycerol: prolipoprotein diacylglyceryl transferase (Lgt) is crucial for immune signaling^5, 22^, we hypothesized that an altered structure of its lipid moiety might be responsible for this surprising phenomenon. Considering that Lpp are TLR2 agonists, we carried out stimulation studies with HEK- and HEK-TLR2 cells, with only the latter being responsive to TLR2 ligands. Again, *S. carnosus* caused an almost 10-times higher IL-8 production than *S. aureus* and *S. epidermidis*, indicating that most likely, Lpp are responsible for the potent immune stimulatory capacity of *S. carnosus*. To confirm a central role of Lpp, we purified His-tagged SitC from *S. carnosus* (SitC-SC) and *S. aureus* (SitC-SA) and applied equal concentrations to HEK-TLR2, with the same result, namely, that SitC-SC caused a much stronger stimulation than SitC-SA. Considering that it is the lipid moiety that reacts with TLR2–TLR1/6 heterodimers, it was evident that the lipid modification of SitC in the two staphylococcal species had to be different. An important clue came from immune stimulation with the synthetic lipopeptides P2C and P3C in HEK-TLR2 cells where P2C also displayed an ~10-fold higher activation of TLR2 than P3C. We, therefore, reasoned that SitC-SC might be diacylated, while SitC-SA was previously shown to be triacylated by Kurokawa et al.^24^ In addition to the two *O*-acylated fatty acids at the diacyl-glyceryl portion, triacylated Lpp carry a long-chain *N*-acyl group at the N terminus of *S*-(diacyl-glyceryl) cysteine residue. The enzyme responsible for the *N*-acylation is an apolipoprotein *N*-acyltransferase, which in Gram-negative bacteria is encoded by the *lnt* gene^33, 58^. Recently, P1-3C was tested whether it could trigger eryptosis^59^. The induction of eryptosis was correlated with increasing degree of acylation and was the highest with P3C. However, the concentration to induce eryptosis was roughly 10 times higher compared to immune stimulation. Nevertheless, this study shows that Lpp not only contribute to immune stimulation but that particularly triacylated Lpp damage at higher concentration and also the host cells, thus aggravating an infection^59^.

Although a *lnt* homologous gene/protein has so far not been identified in *S. aureus*, we postulated that there must be an apolipoprotein *N*-acyltransferase-like enzyme and that the corresponding gene is absent or not functional in *S. carnosus*. If this was the case, one could expect that expression of the *E. coli*-specific *lnt* gene in *S. carnosus* should decrease the immune stimulatory activity of Lpp to the level found in *S. aureus*. Our data corroborated this hypothesis (Fig. 1a), suggesting that the enzymatic modification by an *lnt* analog might represent an important prerequisite for enabling commensalism on human skin and mucosal surfaces.

The next step was to analyze the structure of the lipid moiety of SitC-SA, SitC-SC, SitC-SC*lnt*, and SitC-SE (*S. epidermidis*). It turned out that the lipid moiety of SitC-SA, SitC-SE, and of SitC-SC*lnt* was almost identical. The two diacylglyceryl groups were acylated with long-chain fatty acids (C17 and C15, respectively) and the N terminus of *S*-(diacyl-glyceryl) cysteine residue was also acylated with a long-chain fatty acid (C17). This result indicates that *S. aureus* and *S. epidermidis*, the two commensals, possess an Lnt-like enzyme that catalyzes the same reaction as Lnt in *E. coli*.

In some *S. aureus* strains, diacyl Lpp was mainly produced at pH 5.5 and 6.0 and in stationary growth phase. However, we found in the 16-h cell culture of USA300 only triacylated SitC and Lpl1. A possible reason for the pH effect in *S. aureus* could be that the expression of the gene encoding the apolipoprotein *N*-acyltransferase-like enzyme is induced only under certain conditions. Once the gene is identified, this question will be addressed in more detail in a human skin model. Besides triacylated, diacylated, and the short-chain *N*-acetylated Lpp, there was also a *N*-acyl-*S*-monoacyl-glyceryl-cysteine (named the lyso structure) identified in low-GC Gram-positive *Enterococcus faecalis*, *Bacillus cereus*, *Streptococcus sanguinis*, and *Lactobacillus bulgaricus* ^23^. Recently, the gene leading to the lyso structure has been identified in *Enterococcus faecalis* and *Bacillus cereus* and was referred to as lipoprotein intramolecular transacylase (*lit*)^60^. It was proposed that Lit transfers a fatty acid from the diacylglycerol moiety to the alpha-amino group of the lipidated cysteine. What do we know about other commensal bacteria such as *Propionibacterium* and *Corynebacterium*? Both genera belong to a branch of high % GC Gram-positive bacteria. *Corynebacterium glutamicum* has also a *E. coli*-type Lnt^61^. In propionibacteria, it is unknown, however, that they may also have the *E. coli*-type Lnt.

The most interesting finding, however, was that the N terminus of *S*-(diacyl-glyceryl) cysteine residue in SitC-SC also turned out to be modified, albeit not by a long-chain N-acylation but rather by *N*-acetylation; all other structural elements were identical to those in SitC-SA and SitC-SC*lnt*. This type of *N*-acetylation has, so far, only been described in some *Bacillus* species including *B. subtilis*, *B. licheniformis*, and *B. halodurans*; but it was not detected in *B. cereus*, an environmental bacterial species^23^. The *N*-acetylation of Lpp in *S. carnosus* and the mentioned *Bacillus* species suggest that this reaction is catalyzed by a different enzyme, which can be designated as an apolipoprotein *N*-acetyltransferase (Lat). However, both the enzyme and the acetyl donor are unknown to date. Comparison of immune activation mediated by SitC-SC and P2C was almost indistinguishable, independent of the cell systems used. Thus, SitC-SC behaves like a diacylated Lpp and both SitC-SC and P2C represent strong TLR2/6 agonists. Of note, this further implies that the *N*-acetylation in SitC-SC is most likely too short to allow TLR2/1 specificity and subsequent activation.

Differential activation of TLR2 and its co-receptors influences the cytokine secretion and antigen presentation capacity of innate immune cells^62^. The differences observed in MoDC activation were related to a more powerful induction of IL-6, TNF, and IL-12p40 by SitC-SC than by SitC-SA (Fig. 2c). These cytokines are not only important correlates of TLR2-mediated MoDC activation but they are also important co-stimuli that enhance and shape the adaptive immune response, in particular Th cell differentiation^63^. As proposed by Blander and Medzhitov in 2006, the efficiency of antigen presentation via MHCII relies on the presence of TLR ligands within the phagocytosed matter^64^. In the present case, the intrinsic TLR2 activity of the bacterial lipoprotein SitC enables its phagosomal categorization as a microbial antigen and consecutively drives phagosome maturation and antigen presentation. Furthermore, differential recognition via TLR1 or TLR6 is a fine-tuning element in the regulation of the phagocyte response^62^. It is, therefore, not unexpected that differences in lipid modifications of bacterial proteins that affect the strength and quality of TLR2 activation have direct impact on the efficacy of antigen presentation and subsequent T cell activation. Here, this is demonstrated by the failure of SitC-SA to trigger T cell responses equivalent to those seen with SitC-SC (Fig. 6).

It is known that Th1 and Th17 responses are protective against *S. aureus* and that both Th1 and Th17 cells are important for bacterial clearance^65–67^. Furthermore, it has been proposed that DC-derived cytokines such as IL-6, TNF, IL-1β, TGF-β, and IL-23 are implicated in Th17 cell differentiation^68–70^. Here, we observed that SitC-SC was superior to SitC-SA in triggering a Th1 cell response but there was no evidence for differential induction of Th17 responses (Fig. 6b). Interestingly, an earlier report shows that induction of Th17 responses by MoDC requires engagement of FcγRIIa by bacterial immune complexes (IC) and TLR2 only amplifies this response^71^. The absence of IC formation in our experimental setting might explain the overall low induction of IL-17 secretion from CD4+ T cells.

Notably, the effect of the SitC preparations on the T cell response can be attributed to stimulation of TLR2 and its co-receptors and, additionally, to the presentation of the antigenic peptides derived from the lipoproteins (SitC-SC and SitC-SA) by MoDC. When autologous CD4+ T cells were stimulated with MoDC pulsed with SitC-SC, T cell-derived secretion of IFN-γ (a cytokine specific for Th1 responses) was significantly higher than that with SitC-SA (Fig. 6b). Assuming that lipid modifications will neither affect processing and presentation of the antigen nor the epitopes recognized by T cells, the silencing of co-stimulatory innate immune signals, in particular, the suppression of the Th1-inducing cytokine IL-12^63^ achieved by lipid modifications in SA, represents an important immune evasion mechanism that counteracts the generation of protective T cell responses. This effect was also confirmed in the context of T cell stimulation in PBMC where co-stimulation via SitC-SC and P2C was more effective than that delivered by SitC-SA and P3C (Fig. 6a). Importantly, with the synthetic lipopeptides, this trend was also reproducible in MoDC cocultures with purified T cells but—in contrast to the lipoproteins—the results remained statistically not significant. This may be due to any of three possible reasons: (1) TLR2 itself is not an endocytosis-promoting scavenger receptor. Thus, the stimulatory potential of the synthetic lipopeptides is limited to activation of surface TLR2. By contrast, the potency of the lipoproteins most likely results from additional endosomal TLR activation, as well as antigen processing and presentation to T cells. (2) PBMC contain a broad variety of TLR2-responsive innate immune cells that could compensate for the missing signals in MoDC. (3) T cell stimulation via the TCR and direct co-stimulatory activation of T cell-derived TLR2 might further result in important synergistic effects that cannot be achieved by stimulation with synthetic TLR2 agonists^72^. Well in line with our findings, this effect could be more pronounced on memory T cells when compared to naive T cells (Fig. 6d).

Although *S. carnosus* alerts the immune response, it also leads to cell damage. In the murine tissue cage infection model, we show that the unmodified or short-chain (acetyl) modified N terminus of *S. carnosus* Lpp induced neutrophil cell death and tremendous amounts of TNF. Whether neutrophil death resulted from TLR2 signaling upon recognition of *S. carnosus* Lpp or high levels of TNF as shown in neutrophil, apoptosis^73^ needs to be investigated in further studies. As a consequence of the increased neutrophil death induced by *S. carnosus*, higher CFU were detected in the tissue cage fluid after perioperative infections. On the other hand, *S. aureus*-infected neutrophils can undergo apoptosis and other types of cell death.

Altogether, in our experimental conditions, the induction of a T cell response to Lpp is influenced by its inherent TLR2 activity. Notably, diacylated Lpp such as P2C, or the *N*-acetylated SitC-SC were much more efficient than the triacylated Lpp including P3C, SitC-SA, and SitC-SC*lnt* in mediating the activation of antigen-presenting cells such as MoDC. As a consequence, increased formation of Th1 cells in response to SitC-SC-stimulated MoDC is due to stronger activation of MoDC rather than differences in T cell recognition of the specific antigen (SitC). These results corroborate the importance of innate immune stimulation in shaping adaptive immunity^74^. They imply that modification of lipid structures diminishes innate support of T cell responses and, thus, antibacterial defense. In return, out data suggest that this suppressive action enables host adaptation, persistence, and bacterial commensalism. We can, however, only speculate that Lpp-driven imprintment of innate immunity might even impede the development of protective vaccine-induced immunity.

In conclusion, here, we show that the commensal and noncommensal staphylococcal species differ markedly in immune stimulatory activity. The underlying reason lies, at least partially, in the altered lipid structure of Lpp. In the commensal staphylococcal species, the N terminus of the lipid moiety was found to be modified by a long-chain fatty acid (C17), while the noncommensal *S. carnosus* lipid moiety was modified by a short-chain (C2) fatty acid. The long-chain fatty acid at the N terminus decreased both the innate and adaptive immune responses, thus resulting in immune evasion. We interpret our results as an indication that the commensal species *S. aureus* and *S. epidermidis* are well adapted to the immune system, while *S. carnosus* has not adapted and is, therefore, recognized as foreign and consequently cleared by a fulminant immune response. The molecular basis for the host adaptation in the two commensal species would be an apolipoprotein N-acyltransferase, a Lnt-like enzyme, which has, so far, not been identified. Remarkably, only small structural alterations in the lipid moiety of Lpp hold the balance between immune tolerance and defense. Nevertheless, there is still much to learn on how structural alterations of MAMPs imprint the immune system. While adaptation to the immune system plays an important role in colonization of skin and mucosa, we should not forget that other factors might also promote commensalism. We particularly think of adhesion proteins, capsular polysaccharide, polysaccharide intercellular adhesin (PIA), secreted immune evasion factors, the repertoire of two-component sensor signal transduction systems, etc.

## Methods

### Ethics statement

The use of human peripheral blood mononuclear cells (PBMC) from buffy coats obtained from the German Red Cross South transfusion center (Frankfurt am Main, Germany) was approved by Ethics committee of the Medical Faculty of the Goethe University Frankfurt/Main (Approval #154/15). C57BL/6 mice (janvierLabs, France) were kept under specific pathogen-free conditions in Animal facility of the DBM, University Hospital Basel. Perioperative infections of 13-week-old female mice were performed according to the review board of the Kantonale Veterinaeramt Basel-Stadt (permit no. 1710).

### Bacterial strains and growth conditions

Bacterial strains and plasmids used in this study are listed in Supplementary Table 1. *E. coli* BL21 strain was cultivated aerobically in Luria Broth (LB) medium with shaking at 37 °C. *S. aureus* strains and *S. epidermidis* O47, a biofilm-forming clinical isolate^75^, were aerobically grown in Tryptic Soy Broth (TSB) at 37 °C. To express the gene cloned into the plasmid with inducible xylose promoter, the medium was supplied with 0.25–0.5% xylose in either basic medium, BM without glucose (1% soy peptone, 0.5% yeast extract, 0.5% NaCl, and 0.1% K_2_HPO_4_, pH 7.4), or TSB (TSB: 1.7% tryptone, 0.3% phytone, 0.5% NaCl, 0.25% K_2_HPO_4_, and 0.25% glucose) at 37 °C. To prevent the loss of plasmids during bacteria replication, the media were supplemented with tetracycline (25 μg/ml) for pTX plasmid system or chloramphenicol (10 μg/ml) for pCX and pCtuf plasmid system.

### Construction of *lnt*-expressing plasmid

The *lnt* gene from *E. coli* BL 21 was amplified by using a pair of primers. The forward primer with BamHI cleavage side (underlined sequence), and an optimal Shine–Dalgarno sequence (in italic) with start codon in bold (atataggatcc *aggagg*tattaat**atg**gcttttgcctcattaattgaac). The reverse primer comprised the StreptagII coding sequence (italic sequence), two stop codons (bold sequence), and a XbaI site (underlined sequence) (ttattctaga **ttatta**tttttcaaattgtggatgtgaccatttacgtcgctgacgcagactc). The amplified fragment was ligated in the pCX30 plasmid after digestion with BamHI and XbaI to yield plasmid pCX-*lnt*-strep (Fig. 2a). This plasmid was transformed into *S. carnosus* by electroporation.

### Purification of SitC-his from various staphylococcal clones

The purification of lipoprotein SitC-his from the membrane of *S. aureus* SA113 and *S. carnosus* (pTX30-*sitC*-his), *S. carnosus* (pCX-*lnt*) (pTX30-*sitC*-his), and *S. epidermidis* O47 was carried out following the previous study^76^ with a small modification. Briefly, the clone was precultivated aerobically at 37 °C in 3 l of B medium without glucose until OD_578 nm_ 0.5 was reached, and then the culture was supplemented with 0.5% xylose for 15 h for induction of *sitC* expression. The bacterial cells were harvested by centrifugation at 4000 × *g* at 4 °C for 20 min and washed two times with Tris buffer (20 mM Tris, 100 mM NaCl, pH 8.0). Then, the pellet was resuspended with 100 ml of Tris buffer containing protease inhibitor tablet (Merck, Darmstadt, Germany), DNAse (10 µg/ml), and lysostaphin (30 µg/ml) and incubated at 37 °C for 3 h to disrupt the cell wall. After the first ultracentrifugation (235,000  × *g* for 45 min at 4 °C), membrane proteins were dissolved overnight at 6 °C with Tris buffer containing 2% Triton X100. After another ultracentrifugation step, the supernatant containing the membrane fraction was incubated with 1 ml of Ni-NTA super flow beads (Qiagen, Germany) overnight at 6 °C under mild rotation at 20 rpm. One ml of Ni-NTA beads were washed four times with 20 ml of washing buffer (Tris buffer containing 0.25% Triton X100 and 20 mM imidazole), subsequently the beads were washed two times with 20 ml of the same buffer containing 40 mM imidazole, and finally, the SitC-his was eluted with 10 ml of the same buffer containing 500 mM imidazole. SitC-his was concentrated via centrifugal ultrafilter unit with a molecular mass cutoff of 10 kDa (Sartorius AG, Göttingen, Germany). Purified SitC was verified by SDS-PAGE and Coomassie blue staining, and the concentration of purified SitC was determined by Bradford assay. As excessive Triton X100 interferes with Bradford staining, we diluted our sample 10× with distilled water, thus, the concentration of Triton X100 was only 0.025%, which is compatible with the assay (Biorad manual). To be sure that the SitC concentrations were comparable, we confirmed it again by SDS-PAGE.

### Western blot

Bacteria were harvested by centrifugation at 5000 × *g* for 10 min at 4 °C and washed two times with 20 mM Tris buffer (pH 8). The cell pellets were again dissolved in Tris buffer containing protease inhibitor tablet (Merck, Darmstadt, Germany) and incubated with lysostaphin (30 μg/ml) at 37 °C for 30 min. The membrane proteins were subsequently extracted following the above description, subsequently dissolved in SDS running buffer, and separated in SDS-PAGE. The Lnt-strep was detected by western blot using anti-Strep tag-Antibody (Abcam, Cambridge, England) at the dilution 1:5000.

### Nanoflow liquid chromatography (nLC)–tandem mass spectrometry (MS/MS)

The in-gel digests were analyzed by a nLC–MS/MS consisted of a nanoflow pump with an autoinjector (Easy nLC 1000, Thermo Fisher Scientific, San Jose, CA, USA), a fritless electrospray C4 column (100 μm i.d. × 100-mm length; Nikkyo Technos, Tokyo, Japan), and a quadrupole/Orbitrap hybrid mass spectrometer (Q-Exactive, Thermo Fisher Scientific, San Jose, CA, USA). The method will be described in detail elsewhere. Briefly, the nLC was performed at a flow rate of 300 nl/min using a gradient elution of 0–100% acetonitrile in 0.1% aqueous formic acid solution. The eluate was electrosprayed into the spectrometer which was operated in a data-dependent mode, so that it was automatically switched between MS and MS/MS acquisition. Full scans for surveying precursor ions were acquired with a mass resolution of 70,000 at *m/z* 400. Each of the 10 most intense peaks in a survey scan was isolated within a 4.0-*m/z* window and fragmented by higher-energy collisional dissociation with a normalized collision energy of 30. MS/MS spectra were acquired with a mass resolution of 17,500 at *m/z* 400. The fixed starting mass value for MS/MS was 100.

### HEK-TLR2 stimulation assay

Human embryonic kidney (HEK 293) cells, stably transfected with the human TLR2 gene, were purchased from Invivogen. The cultivation was performed following the previous study^34^. For the bacterial stimulation assay, HEK-TLR2 cells were seeded with 5 × 10^4^ cells/200 μl/well into 96-well cell culture plates and incubated at 37 °C with 5% CO_2_. After 1-day incubation, the number of HEK-TLR2 cells was counted for the stimulation test. Prior to stimulation, bacteria were grown in TSB for 6 h. The cells were harvested and washed three times with DMEM/F before measuring the OD_578_ in DMEM/F. To calculate bacterial dosage (MOI, multiplicity of infection), bacteria were set to the distinct OD/CFU. For *S. aureus* USA300 and SA113, OD_578_ of 1.0 equates to 1 × 10^8^ CFU (colony- forming units)/ml, however, for HG003 and *S. carnosus*, it equates to 0.4 and 0.2 1 × 10^8^ CFU/ml, respectively. The final bacterial dosage (MOI 10) was suspended in 50 μl of the HEK-TLR2 medium for the stimulation. Bacteria were unable to grow due to antibiotic supplementation in stimulation medium. For the stimulation assays with purified proteins, the proteins were diluted in the HEK-TLR2 medium into three different amounts (10, 20, and 50 ng) into 200-μl medium containing around 1 × 10^5^ HEK-TLR2 cells prior to application for the test. Two synthetic lipopeptides, P3C (Pam_3_CSK_4_) and P2C (Pam_2_CSK_4_), were purchased from EMC (Tübingen, Germany), dissolved in water, and used for 100 ng for the above volume stimulation test, equivalent to the concentration of 500 ng/ml. Stimulation was carried out for 18 h. The supernatants were collected and stored at −20 °C until use.

For expression of TLR1/TLR2 and TLR6/TLR2 heterodimers in HEK293 cells, HEK293 cells were seeded at 5 × 10^4^/200-µl RPMI (Gibco, Life science, Darmstadt, Germany) containing 10% fetal calf serum (FCS) (Sigma-Aldrich Chemie GmbH, Munich, Germany), 1% penicillin/streptomycin, and 1% l-glutamine (both from Biochrom, Berlin, Germany) and incubated overnight at 37 °C, 5% CO_2_. The next day, the medium was replaced with 150-µl OptiMEM (Gibco, Life science, Darmstadt, Germany). Transient transfection was performed by complexing 0.25-µl lipofectamine 2000 (Invitrogen, Karlsruhe, Germany) with plasmid (pFLAG-CMV-1) encoding TLR1, TLR2, or TLR6. The total amount of plasmid DNA in the 50-µl OptiMEM medium used per well for transfection was 100 ng for single TLR1/2/6 or 90 ng of TLR1 or –TLR6 plasmid plus 10 ng of TLR2 plasmid only to avoid recognition of Lpp by TLR2 only. Lipofectamine (LF) alone and untransfected HEK293 cells were used as controls. After overnight incubation, cells were washed and stimulated with 100 ng/ml SitC-SA, SitC-SC, P2C, or P3C. After 18 h, supernatants were collected and stored at −20 °C.

### MonoMac6 stimulation assay

MonoMac6 (MM6), a human monocytic leukemia cell line, was obtained from DSMZ with number AAC 124 (Braunschweig, Germany) and cultured as previously described^37^. For the stimulation assay, MM6 cells were seeded 1 × 10^6^ cells/1 ml/well into 24-well cell culture plates and incubated for 1 h at 37 °C with 5% CO_2_. The MOI (ratio of bacteria vs. MM6 cells) was 30. For the stimulation assays with purified SitC, three concentrations were applied (50, 100, and 250 ng); as control, P3C and P2C were applied at a concentration of 200 ng. MM6 stimulation was carried out for 4 h; subsequently, the supernatants were collected and stored at −20 °C until they were used for ELISA.

### Generation of monocyte-derived dendritic cells

Buffy coats of healthy donors were obtained from German Red Cross South transfusion center (Frankfurt am Main, Germany). Informed consent was obtained from all donors. The use of the buffy coat cells was approved by ethics committee of the Medical Faculty of the Goethe University Frankfurt/Main (Approval #154/15). PBMC were isolated by Pancoll gradient centrifugation (PAN-Biotech, Aidenbach, Germany). Monocytes were isolated by positive selection with anti-CD14 microbeads (Miltenyi Biotech, Bergisch-Gladbach, Germany). The purity was analyzed by flow cytometry on a FACS LSRII SORP (BD Biosciences, Heidelberg, Germany) with anti-human CD14 V450 (BD Biosciences, Heidelberg, Germany) and ranged from 90% to 99%. The remaining PBMC were frozen in RPMI 1640 supplemented with 20% FCS and 20% DMSO (both from Sigma-Aldrich, Munich, Germany) for subsequent isolation of autologous T cells.

Isolated monocytes were seeded at a density of 1.5 × 10^6^ cells/ml in RPMI 1640 (Gibco, Life science, Darmstadt, Germany), supplemented with 10% FCS (Sigma-Aldrich Chemie GmbH, Munich, Germany), 1% penicillin/streptomycin, 1% l-Glutamine and 1% HEPES buffer (all from Biochrom, Berlin, Germany), 50 µM 2-Mercaptoethanol (Sigma-Aldrich, Munich, Germany), 50 ng/ml human GM-CSF, and 20 ng/ml human lL-4 (both from Miltenyi Biotech, Bergisch-Gladbach, Germany). Cells were incubated at 37 °C and 5% CO_2_, medium exchanged after 3 days, and cells were harvested on day 6. Differentiation of monocytes into MoDC was verified by flow cytometric analysis using anti-CD14-V450, anti-CD83-APC, anti-CD11c-PE, and anti-HLA-DR-PerCP-Cy5.5 (all from BD Biosciences, Heidelberg, Germany) for a CD14^−^, HLA-DR^high^, CD11c^+^, and CD83^dim^ phenotype.

### Stimulation and transfection of MoDC and coculture experiments with autologous T cells or PBMC

For isolation of autologous T cells, frozen PBMC were thawed and total CD4+ T cells were isolated via positive selection with anti-CD4 microbeads, respectively (Miltenyi Biotech, Bergisch-Gladbach, Germany). The purity was analyzed by flow cytometry with anti-human CD4-PerCP-Cy 5.5 and anti-CD8-PE (both from BD Biosciences, Heidelberg, Germany) and ranged from 85% to 98%. Naive and memory T cell fractions were sorted on a FACSAria Fusion (BD Biosciences, Heidelberg, Germany) using BD FACS Diva software version 8.0.1. To this end, purified CD4+ T cells were labeled with anti-CD8 APC, anti-CD14 V450, anti-CD45RA PE, and anti-CD45RO APC-H7 (all from BD Biosciences). The sorting scheme is shown in Fig. 6c. Purity of sorted subpopulations was confirmed by remeasuring of samples after sorting (≥99%). Sorted cells were washed, counted, and checked for viability using trypan blue (Applichem Panreac, Darmstadt, Germany).

MoDC were harvested on day 6 of culture by washing cells vigorously with DPBS (Gibco, Life science, Darmstadt, Germany). MoDC were seeded at a density of 1 × 10^5^/ml RPMI 1640 (Gibco by Life science, Darmstadt, Germany), supplemented with 1% penicillin/streptomycin (10.000 IU/ml and 10.000 μg/ml), 1% 200 mM l-Glutamine (all from Biochrom AG, Berlin, Germany,) and 10% FCS (Sigma-Aldrich) and stimulated with 0.1 µg/ml SitC purified from *S. aureus* (SitC-SA) or *S. carnosus* (SitC-SC), or stimulated with 0.1 μg/ml of P2C, P3C, or nonstimulatory P1C (all from EMC, Tübingen, Germany). For PBMC experiments, MoDC were prestimulated with TLR2 ligands (0.1 μg/ml) and added to autologous PBMC at a ratio of 1:5 after 2.5 h. For cocultures with autologous T cells, MoDC were pulsed with 0.1 µg/ml SitC purified from *S. aureus* and *S. carnosus*, respectively, or stimulated with 0.1 or 1 μg/ml of the synthetic TLR2 agonists P2C, P3C, or nonstimulatory P1C. CD4^+^ T cells were added to MoDC at a ratio of 5:1, and cocultures were incubated at 37 °C and 5% CO_2_ for the indicated time periods.

### Mice and tissue cage infection model

C57BL/6 mice (janvierLabs, France) were kept under specific pathogen-free conditions in Animal facility of the DBM, University Hospital Basel. Perioperative infections of 13-week-old female mice were performed according to the regulations of the Swiss veterinary law (permit no. 1710). As described earlier^39^, sterile Teflon cages (8.5 by 1 by 30 mm; volume 1.9 ml, 130 regularly spaced holes) (Angst+Pfister AG, Zurich, Switzerland) were implanted subcutaneously into the flanks of mice. After wound closure, 10^9^ cfu of either *S. carnosus* TM300 or *S. carnosus* pCX-*lnt* were injected percutanously into the Teflon cages. Tissue cage fluid (TCF) was sampled on day 2 and 6 after infection to quantify (1) viability and number of leukocytes with ADAM-MC (NanoEnTek), (2) bacterial load by plating serial dilution of TCF on MHB agar plates, and (3) tumor necrosis factor (TNF) and interleukin 6 (IL-6) in the TCF supernatant by ELISA (Ready-Set-Go!®, eBioscience).

### Detection of cytokines

*ELISA*. Human cytokine secretion was measured in cellular supernatants using the BD OptEIA ELISA kits for IL-1β, IL-4, IL-6, IL-8, IL-10, IL-12p40, IFNγ and TNFα, and IL-17A ELISA from R&D Systems (Minneapolis, MN, USA) according to the manufacturer´s instructions.

*ELISpot*. For ELISpot assays, MoDC/T cell cocultures were performed in 96-well MultiScreen HTS IP plates (0.45 µm, clear, Merck Chemicals GmbH, Darmstadt, Germany), coated with anti-IFNγ capture antibody (BD Biosciences, Heidelberg, Germany) overnight at 4 °C. Following blocking of the plate for 2 h at room temperature with culture medium, cells were seeded and stimulated as described above. After 16 h of incubation, cells were discarded and wells were washed with sterile water two times followed by washing with PBS/0.05% Tween20 (Sigma-Aldrich Chemie GmbH, Munich, Germany) three times. Biotinylated anti-human IFNγ detection antibody (BD Biosciences, Heidelberg, Germany) was added in PBS/10% FCS and plates incubated for 2 h. After washing in PBS/0.05% Tween20, Alkaline Phosphatase (AP)-conjugated Streptavidin (BD Biosciences, Heidelberg, Germany) was added 1:1000 in PBS/10% FCS followed by incubation for 1 h. Development of the plate was performed with the AP conjugate substrate kit (Bio-Rad Laboratories GmbH, München, Germany); the reaction was stopped by washing with water and the plate dried overnight. Spots and enzymatic activity were quantified with an iSpot FluoroSpot Reader System (AID, Strassberg, Germany).

### Endotoxin testing

Purified SitC-SA and SitC-SC were tested for endotoxin contamination using the Endosafe-PTS System (Charles River, USA). Endotoxin levels for 1 µg/ml protein solutions were <0.005 EU/ml for SitC-SA and 0.006 EU/ml for SitC-SC.

### Statistical analysis

Unpaired two-tailed Student’s *t* test or analysis of variance (one-way ANOVA) was used to compare the difference of means. Statistical analysis was performed using SPSS v.19. For the Luminex analysis in MoDC, Wilcoxon matched- pairs signed rank test as a nonparametric version of the dependent t test was applied. For in vivo experiments, mouse values are shown as mean value ± s.d. of each group. Mann–Whitney test was used for comparison of CFU, leukocyte number and viability, TNF, and IL-6 concentrations in TCF from mice infected with *S. carnosus* and *lnt*. The significant level was set as: not significant (ns) *P* > 0.05; *P < 0.05; **P < 0.01; ***P < 0.001, ****P < 0.0001.

### Data availability

All the relevant data supporting the findings of the study are available in this article and its Supplementary Information files, or from the corresponding author upon request.

## Electronic supplementary material

Supplementary Information
