# Supplementary material for: Lipid moieties on lipoproteins of commensal and non-commensal staphylococci induce differential immune responses
Source: Nat Commun. 2017 Dec 21;8:2246. doi: 10.1038/s41467-017-02234-4 (PMC5740139; doi:10.1038/s41467-017-02234-4)
Supplement: Supplementary file 1 — Supplementary Information [file 41467_2017_2234_MOESM1_ESM.pdf]

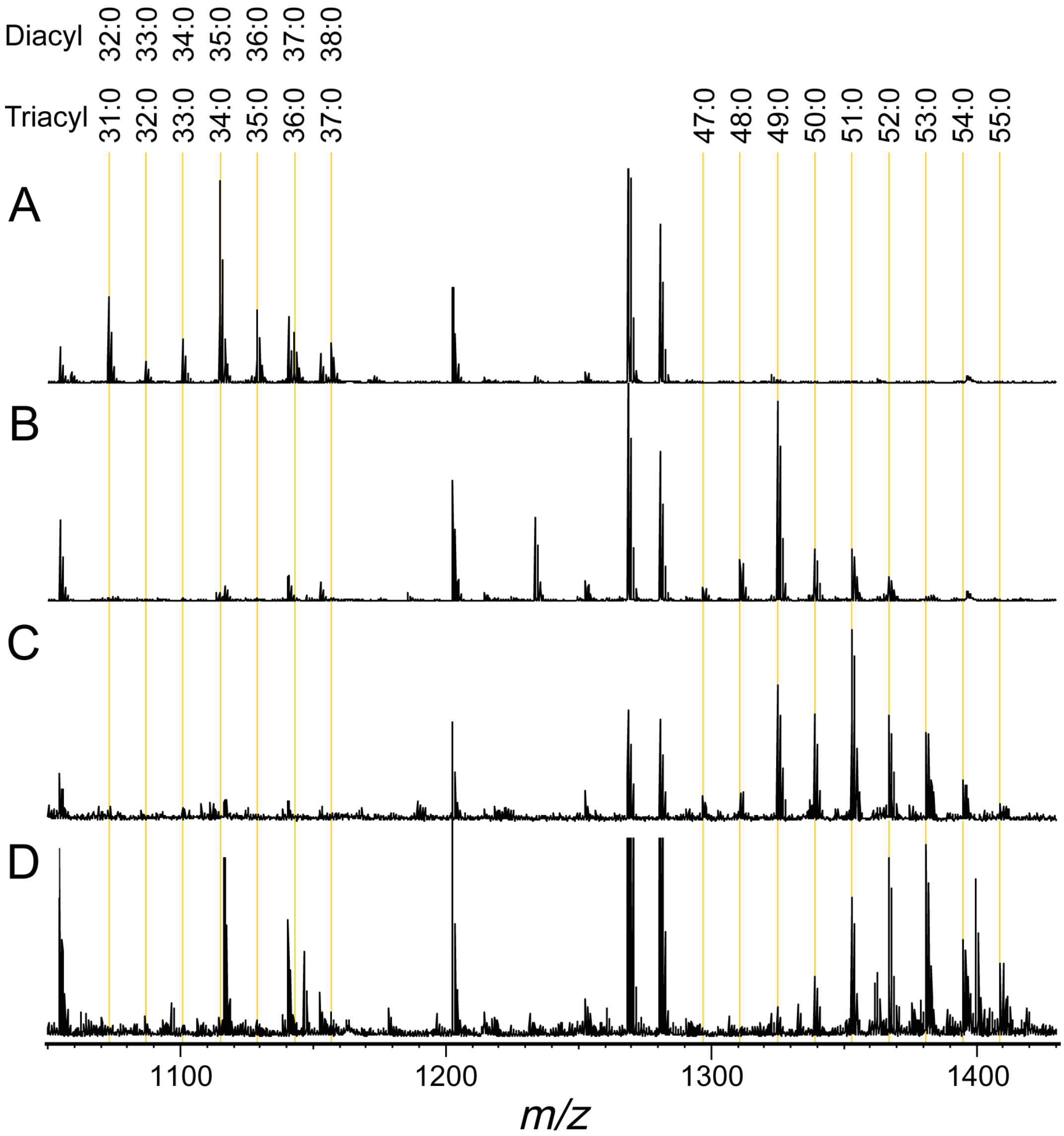

Supplementary Figure 1. The mass distributions of the N-terminal tryptic peptides of the SitC proteins isolated from various staphylococci. MALDI TOF MS spectra of in-gel tryptic digests of SitC-SC (A), SitC-SC*Int* (B), SitC-SA (C) and SitC-SE (D) are shown. The 14-Da mass differences in mass signals corresponding to methylene group are due to the *N*- and/or *O*-acylations with various lengths of fatty acids. The numbers of total carbon atoms and double bonds in fatty acyl groups of possible diacyl and triacyl lipopeptides are labelled. Peaks that are detected commonly between *m/z* 1200 and 1290 are SitC-derived non-lipidated peptides.

A

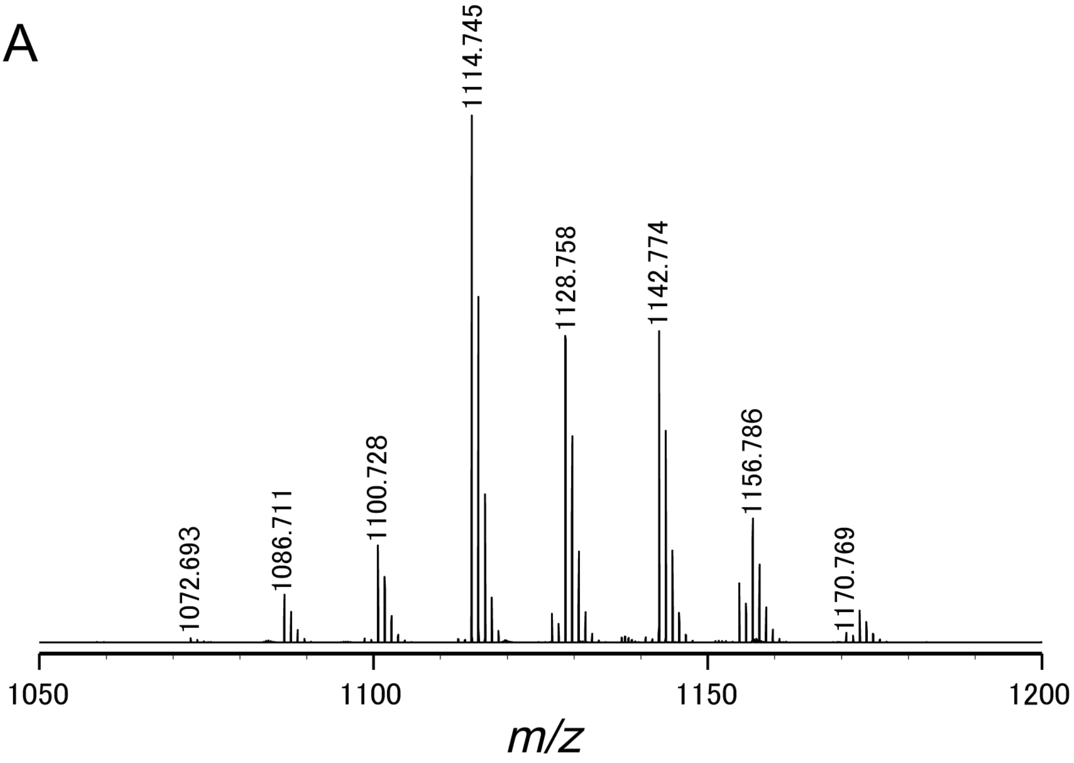

B

Observed *m/z*	Diacyl Carbon number	Diacyl Calculated *m/z*	Diacyl relative error (ppm)	Triacyl Carbon number	Triacyl Calculated *m/z*	Triacyl relative error (ppm)
1072.693	32	1072.731	-35	31	1072.694	-1.4
1086.711	33	1086.746	-32	32	1086.710	1.2
1100.728	34	1100.762	-31	33	1100.726	2.1
1114.745	35	1114.778	-29	34	1114.741	3.7
1128.758	36	1128.793	-31	35	1128.757	1.3
1142.774	37	1142.809	-30	36	1142.773	1.6
1156.786	38	1156.825	-33	37	1156.788	-2.0
1170.769	39	1170.840	-61	38	1170.804	-30

Supplementary Figure 2. The accurate mass measurement reveals the triacyl structure of SitC-SC. (A) nLC-MS spectrum of the N-terminal lipopeptides of SitC-SC. **(B)** A table that represents observed and calculated mass values and assignments of the lipopetide peaks detected in A. The mass errors indicate that those lipopeptides are triacylated.

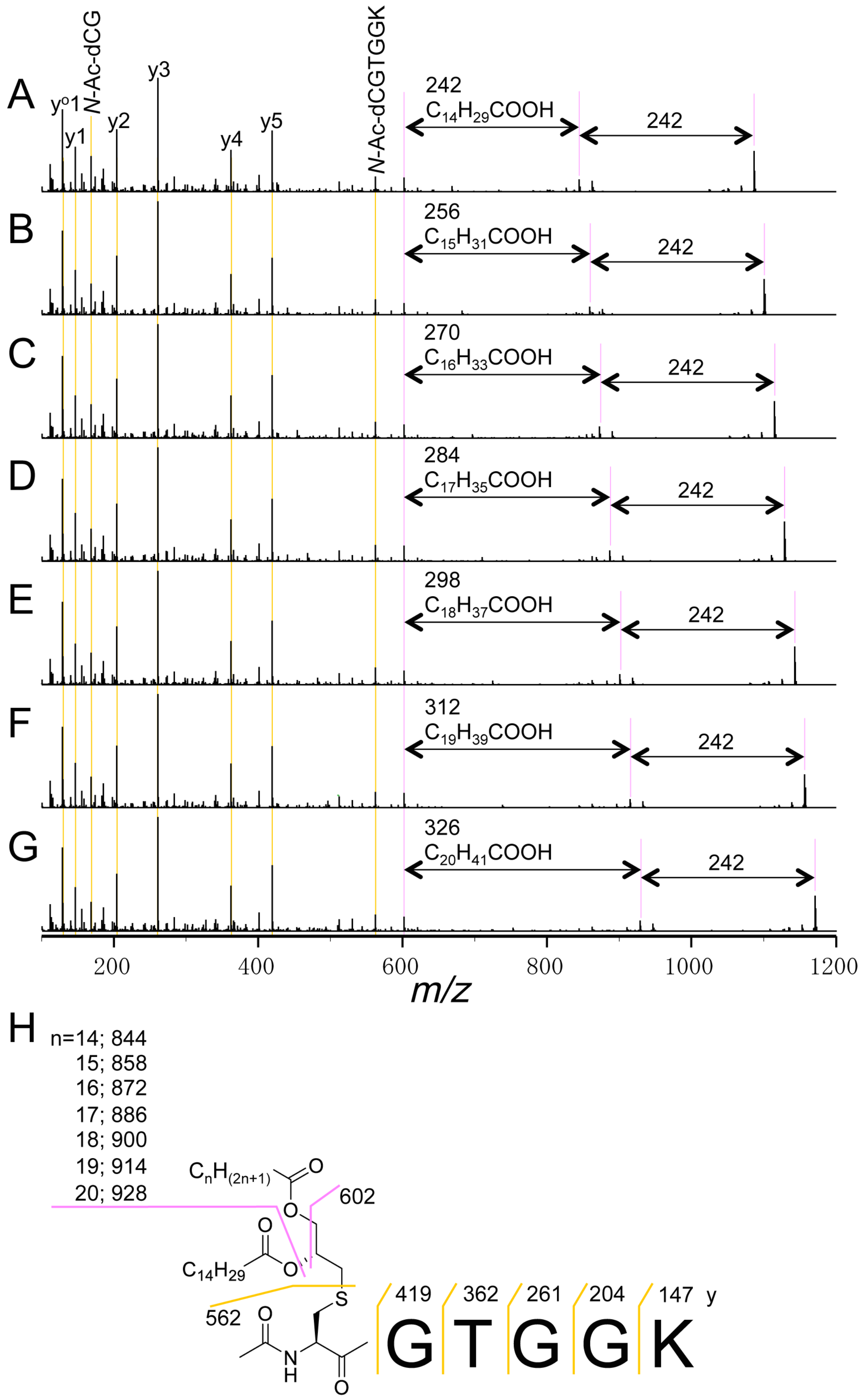
A
B
C
D
E
F
G
H
y°1
y1
N-Ac-dCG
y2
y3
y4
y5
N-Ac-dCGTGGK
242
C14H29COOH
256
C15H31COOH
270
C16H33COOH
284
C17H35COOH
298
C18H37COOH
312
C19H39COOH
326
C20H41COOH
200
400
600
800
1000
1200
m/z
n=14; 844
15; 858
16; 872
17; 886
18; 900
19; 914
20; 928
CnH(2n+1)
602
C14H29
562
419
362
261
204
147
y
GTGGK

Supplementary Figure 3. The lipid part of SitC-SC is α*N*-acetylated. nLC-MS/MS spectrum of the N-terminal lipopeptide of SitC-SC at *m/z* 1086.7 **(A)**, 1100.7 **(B)**, 1114.7 **(C)**, 1128.8 **(D)**, 1142.8 **(E)**, 1156.8 **(F)** or 1170.8 **(G)** corresponding to triacyl forms (31:0, 32:0, 33:0, 34:0, 35:0, 36:0, 37:0 or 38:0 in total, respectively). **(H)** The elucidated α*N*-acetyl structure of SitC-SC. The C-terminus-containing y-ion series that confirmed the GTGGK sequence and product ions that have lost pentadecanoic acid, two fatty acids or diacylpropanediols from the molecular ion are depicted in the spectra. y^{o} denotes dehydrated y ion. dC: dehydrocysteine.

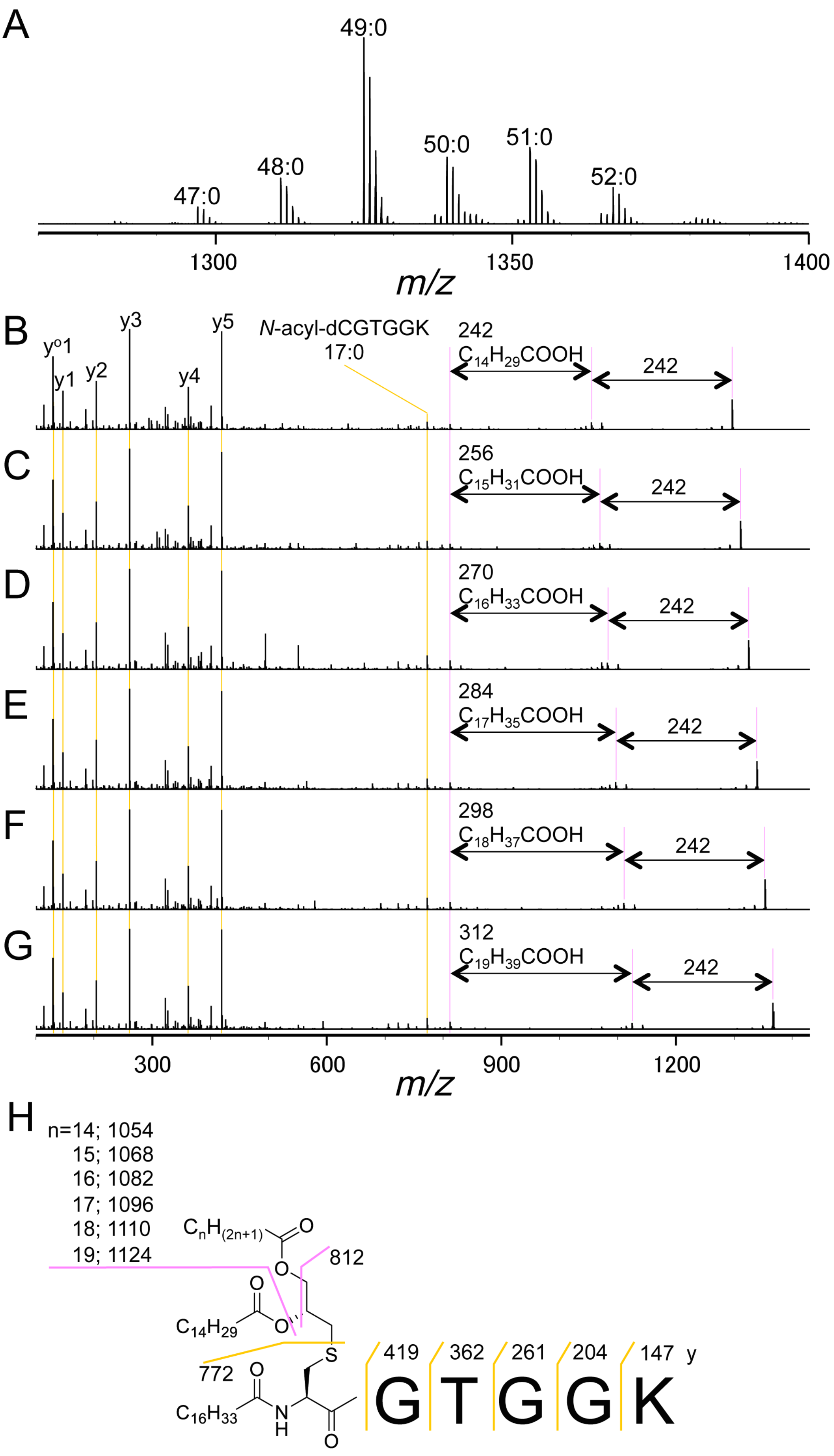

A
49:0
48:0
47:0
50:0
51:0
52:0
1300
1350
1400
m/z
B
y°1
y1
y2
y3
y4
y5
N-acyl-dCGTGGK
17:0
242
C14H29COOH
242
C
256
C15H31COOH
242
D
270
C16H33COOH
242
E
284
C17H35COOH
242
F
298
C18H37COOH
242
G
312
C19H39COOH
242
300
600
900
1200
m/z
H
n=14; 1054
15; 1068
16; 1082
17; 1096
18; 1110
19; 1124
CnH(2n+1)
812
C14H29
772
C16H33
419
362
261
204
147
y
GTGGK

Supplementary Figure 4. The SitC-SC*Int* is α*N*-heptadecanoylated. (A) nLC-MS spectrum of the N-terminal lipopeptide of SitC-SC*Int.* The major peaks of the lipopeptide are labelled with the total numbers of carbon atoms and double bonds of their fatty acyl groups. **(B~G)** nLC-MS/MS spectrum of each peak in A at *m/z* 1296.9 **(B)**, 1311.0 **(C)**, 1325.0 **(D)**, 1339.0 **(E)**, 1353.0 **(F)** or 1367.0 **(G)** corresponding to triacyl form (47:0, 48:0, 49:0, 50:0, 51:0 or 52:0 in total, respctively) is shown. **(H)** The elucidated α*N*-heptadecanoylated triacyl structure of SitC-SC*Int*. The C-terminus-containing y-ion series that confirmed the GTGGK sequence and product ions that have lost pentadecanoic acid, two fatty acids (30:0~35:0 in total) or diacylpropanediol from the molecular ion are depicted. y^{o} denotes dehydrated y ion. dC: dehydrocysteine.

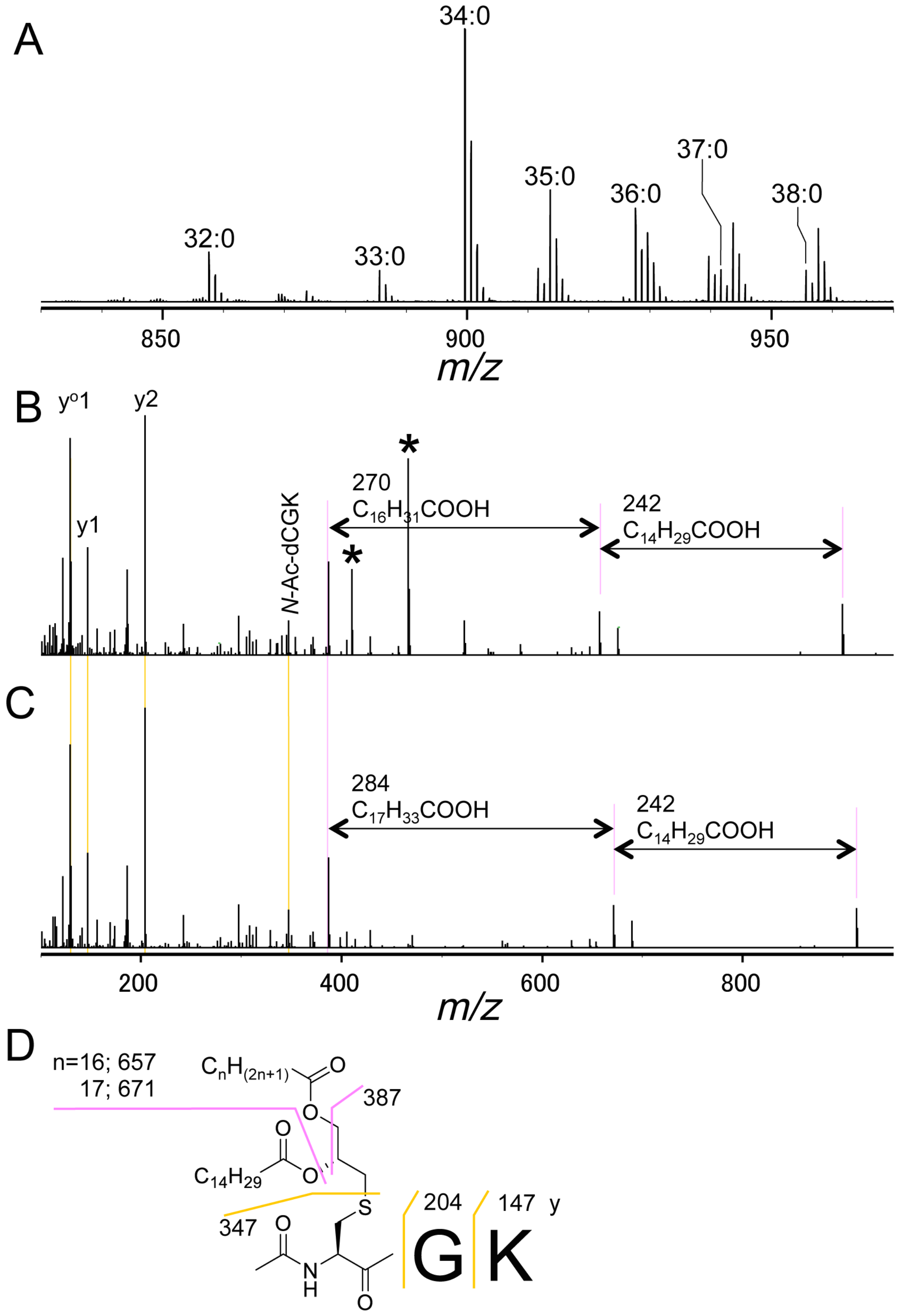

Supplementary Figure 5. The Lpl1-SC protein is also α*N*-acetylated. (A) nLC-MS spectrum of the N-terminal lipopeptide of Lpl1-SC. Most intense peaks are identified as triacyl form based on the accurate mass measurement. Only the peak at *m/z* 857.6 was diacylated. The total numbers

of carbon atoms and double bonds are marked in the figure. **(B,C)** The nLC-MS/MS spectrum of the peak at *m/z* 899.65 **(B)** or 913.67 **(C)** corresponding to triacyl form (34:0 or 35:0 in total, respctively) is shown. **(D)** The elucidated α*N*-acetyl structure of Lpl1-SC. The C-terminus-containing y-ion series that confirmed the GK sequence and product ions that have lost pentadecanoic acid, two fatty acids (32:0 or 33:0 in total) or diacyl(32:0 or 33:0)propanediol from the molecular ion are depicted. y^{o} denotes dehydrated y ion. dC: dehydrocysteine. Asterisks denote contamination signals.

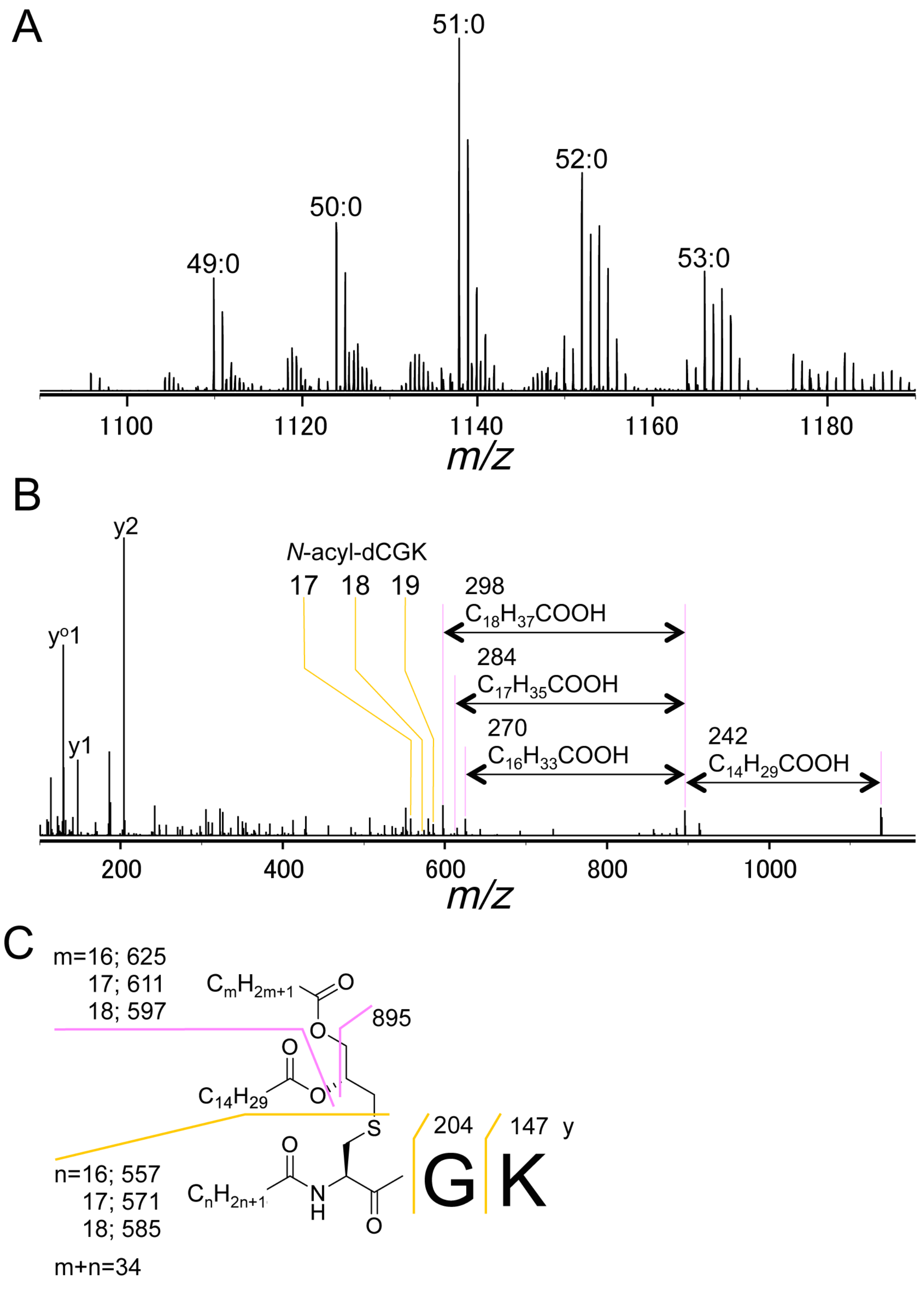

Supplementary Figure 6. The Lpl1-SA protein is also α*N*-long-chian-acylated. (A) nLC-MS spectrum of the N-terminal lipopeptide of Lpl1-SA. The total numbers of carbon atoms and double bonds are marked in the figure. **(B)** The nLC-MS/MS spectrum of the peak at *m/z* 1137.92 (51:0 in total) is shown.

(C) The elucidated α*N*-long-chain-acyl structure of Lpl1-SA. The C-terminus-containing y-ion series that confirmed the GK sequence and product ions that have lost pentadecanoic acid, two fatty acids or diacylpropanediol from the molecular ion are depicted. Triacyl form acylated with 17:0-, 15:0- and 19:0-fatty acids at *sn*-1, *sn*-2 and α-amino group, respectively, and that with 19:0, 15:0 and 17:0 are predominant molecular species. y^o denotes dehydrated y ion. dC: dehydrocysteine.

Supplementary Table 1. List of bacterial strains and plasmids used in the study

Strains or plasmids	Descriptions	Reference
**Bacterial strains**		
E. coli BL21	Laboratory strain	
S. carnosus TM300	Laboratory strain	1
S. aureus 113	Mutant of RN1 (NTCC8325), r^- m^-, agr^-, $sigB^-$	2
S. aureus HG003	Mutant of strain RN1 (NTCC8325), *rsbU&tcaR* repaired	3
S. aureus USA300 JE2	Cured from the three plasmids present in the parent strain USA300 LAC	4
S. epidermidis O47	Biofilm-forming clinical isolate	5
**Plasmids**		
pCX30	Xylose inducible expression vector encoded a hybrid lipase-FnBPB	6
pCX-*lnt*-strep	Express $Lnt^{E.c}$ under xylose inducible promoter	This study
pTX-*sitC*-his	Express full length SitC-his under xylose inducible promoter	7

Supplementary References

1. Rosenstein R*, et al.* Genome analysis of the meat starter culture bacterium *Staphylococcus carnosus* TM300. *Appl Environ Microbiol* **75**, 811-822 (2009).

2. Iordanescu S, Surdeanu M. Two restriction and modification systems in *Staphylococcus aureus* NCTC8325. *J Gen Microbiol* **96**, 277-281 (1976).

3. Herbert S*, et al.* Repair of global regulators in *Staphylococcus aureus* 8325 and comparative analysis with other clinical isolates. *InfectImmun* **78**, 2877-2889 (2010).

4. Diep BA*, et al.* Complete genome sequence of USA300, an epidemic clone of community-acquired meticillin-resistant *Staphylococcus aureus*. *Lancet* **367**, 731-739 (2006).

5. Heilmann C, Gerke C, Perdreau-Remington F, Götz F. Characterization of Tn917 insertion mutants of *Staphylococcus epidermidis* affected in biofilm formation. *Infect Immun* **64**, 277-282 (1996).

6. Strauss A, Götz F. In vivo immobilization of enzymatically active polypeptides on the cell surface of *Staphylococcus carnosus*. *Mol Microbiol* **21**, 491-500 (1996).

7. Müller P*, et al.* The *Staphylococcus aureus* lipoprotein SitC colocalizes with Toll-like receptor 2 (TLR2) in murine keratinocytes and elicits intracellular TLR2 accumulation. *Infect Immun* **78**, 4243-4250 (2010).
